# Rubber Bands in the Treatment of Fractures

**Published:** 1873-05

**Authors:** J. W. Southworth

**Affiliations:** Toledo, O.


ART III.—*Elastic Rubber Bands in the treatment of Fractures.* Notes by J. W. SOUTHWORTH, M. D., Toledo, O.

The fact that sections of rubber tubing are used for making extension in case of fractured or contracted limbs, and in the deformities of club-foot, is now well known and often taken advantage of by the profession. The elastic rubber stocking for compressing varicose veins, is also familiar. Perforated rubber tissue bandages are being introduced as a substitute for common dressings, as well as for the purpose of making continuous compression in the various enlargements or swellings of the limbs or joints. These make the amount and extent of pressure most easily and perfectly within the control of the surgeon. An advantage which all must concede to be of no little moment.

There are few of us, I apprehend, who have not unfortunately found after a first or subsequent dressing of a broken limb, that the straps had become loosened, the splints and the fragments of bone displaced, which were so carefully adjusted, and treated *secundem artem.* This misfortune we have often, no doubt, very justly attributed to the imperfection of the means at our command; though sometimes very properly, to the refractory or careless disposition of the patient, this being most common in young subjects, whom by the way, it is more imperatively the part of the physician to "cure" with as perfect and useful a limb as possible. Such a desideratum, I am happy to state, from personal experience, is attainable by the substitution of elastic retention bands in lieu of the ordinary inelastic cloth bands or bandages, or straps of webbing. These elastic straps are most promptly improvised by taking the common rubber bands (from one-quarter to one-half inch in width, by two inches in length) found in the book or drug stores; doubling them, and passing strips of strong muslin or factory cloth through the doubled band so as to make it a part of the strap; thus allowing it to be stretched to the extent deemed advisable to produce the requisite degree of constricting force when applied around the splints.

In fractures of the fore-arm treated with two lateral splints, four such straps usually suffice for grown persons, and for children also; but in them the smaller sized bands (doubled) are to be used. In fractures of the leg or thigh more will be necessary, of course. Where two parallel lateral splints are used, as in fractures of the fore-arm, the rubber portion of the encircling straps must be placed between the opposing splints alternately on the superior and inferior borders, so as to counterpoise or preserve the balance of the constricting forces; and in cases of the arm, leg or thigh where more splints are used the rubber part of the straps should be likewise adapted to the interspaces of the splints, in order to attain the same object as nearly as possible.

By these means a sufficient amount of retentive force is *constantly* in operation, and if much swelling takes place there will be a conservative yielding of the encircling bands, which is not the case where cloth, webbing, or leather straps are used. Also, when the swelling subsides, no matter *how rapidly*, there is always a coincident as well as a commensurate adaptation to the diminished size of the limb through the agency of the rubber; thus preventing as far as possible an accident, so much to be avoided in case of restless or refractory children, or even in older subjects. As an after-dressing, when osseous union has taken place and nothing but a precautionary use of splints is required, the use of these elastic bands or straps around either sole leather, paste board, or felt splints, is the most perfect dressing, in my estimation, yet devised. I am quite sure that those who resort to their application will not dissent from such conclusion.

If either of the above materials are used it will be found best to apply around them (after they have been softened with hot water) an ordinary roller, or a many-tailed bandage of cotton or flannel at first, substituting the elastic straps the next day or two, when the splints are well dried and set. In order to fix the splints quite immovably on the skin, a strap of adhesive plaster two inches wide and as long as the splint, may be placed lengthwise along the middle of it, with the non-adhering surface against the concave or inner side of the same; applying a short strip about an inch wide over and crossways of the long one, at the middle and at each end, so as to secure it firmly; thus affording an adhesive surface which will cause the splints to remain in situation most admirably after they are applied to the limb. This principle can be adapted to almost any form of splints, especially pasteboard, covered with cloth or not; to sole leather, felt, etc.

The most eligible, cheap and efficient after-dressing that I have ever tried, is the pasteboard covered with canton flannel pasted on (with the nap out),—so as to completely envelope the splints,—with thick starch paste immediately previous to their application; the roller or a many-tailed bandage being used until the splints set dry, then arranging the adhesive plaster as detailed above, using the elastic straps for retention afterwards. The pasteboard should be of the heavy "book-board" kind, two pieces being used for the fore-arm, arm, and leg; and three for the thigh. It should also be dipped in water before the cloth is pasted on, so as to render it sufficiently pliable to be moulded by the hands approximately to the contour of the limb as the cloth is put on; the bandage then being simply applied will complete the adaptation most perfectly.

It is of course understood that proper support by bandages will be given the injured limb below the seat of fracture, or at least up to the distal ends of the splints. By this plan we may bid good bye to the cumbersome plaster of paris after-dressing for all ordinary cases and circumstances.

